# Vitamin D supplementation in children with asthma: a systematic review and meta-analysis

**DOI:** 10.1186/s13104-014-0961-3

**Published:** 2015-02-03

**Authors:** Munes M Fares, Lina H Alkhaled, Salman M Mroueh, Elie A Akl

**Affiliations:** Department of Pediatrics and Adolescent Medicine, American University of Beirut, Beirut, Lebanon; Department of Internal Medicine, American University of Beirut, Beirut, Lebanon; Department of Clinical Epidemiology and Biostatistics, McMaster University, Hamilton, ON Canada; Department of Medicine, State University of New York at Buffalo, Buffalo, NY USA

**Keywords:** Asthma, Allergy, Atopy, Vitamin D, Cholecalciferol, Calcitriol, Ergocalciferol, Children

## Abstract

**Background:**

Epidemiologic studies suggest an association between vitamin D deficiency and atopic diseases, including asthma. The objective of this study was to systematically review the benefits and harms of vitamin D supplementation in children with asthma.

**Methods:**

We used standard Cochrane systematic review methodology. The search strategy included an electronic search in February 2013 of MEDLINE and EMBASE. Two reviewers completed in duplicate and independently study selection, data abstraction, and assessment of risk of bias. We pooled the results of trials using a random-effects model. We assessed the quality of evidence by outcome using the GRADE methodology.

**Results:**

Four trials with a total of 149 children met eligibility criteria. The trials had major methodological limitations. Given the four studies reporting on asthma symptoms used different instruments to measure that outcome, we opted not to conduct a meta-analysis. Three of those studies reported improvement in asthma symptoms in the vitamin D supplemented group study, while the fourth reported no effect (very low quality evidence). For the lung function outcome, a meta-analysis of two trials assessing post treatment FEV-1 found a mean difference of 0.54 liters per second (95% CI -5.28; 4.19; low quality evidence). For the vitamin D level outcome, a meta-analysis of three trials found a mean difference of 6.56 ng/ml (95% CI -0.64; 13.77; very low quality evidence).

**Conclusions:**

The available very low to low quality evidence does not confirm or rule out beneficial effects of vitamin D supplementation in children with asthma. Large-scale, well-designed and executed randomized controlled trials are needed to better understand the effectiveness and safety of vitamin D in children with asthma.

**Electronic supplementary material:**

The online version of this article (doi:10.1186/s13104-014-0961-3) contains supplementary material, which is available to authorized users.

## Background

Asthma is the most common chronic disease of childhood. Its prevalence has been steadily increasing worldwide over the past few decades, along with that of atopic diseases in general. This has been most apparent in the developed countries [1-3]. The reasons for this increase have not been well defined and are the subject of extensive research. They are thought to include changes in environment and lifestyle, including nutritional patterns [4].

Of the nutrients that have been studied, vitamin D has received particular attention. Besides its known effects on bone metabolism, vitamin D seems to play a number of other roles in the body, including an important immunoregulatory function [5,6]. Experimental and epidemiologic studies have tried to establish an association between vitamin D and asthma and atopic diseases. The bulk of the evidence suggested a protective effect [7-9], although some reports did show a deleterious effect of vitamin D on atopic diseases [10,11]. A number of interventional studies have been undertaken or are currently underway to assess the effect of vitamin D supplementation on asthma.

The objective of this study was to systematically review the benefits and harms of vitamin D supplementation in children with asthma.

## Methods

### Protocol and registration

We registered the systematic review protocol with PROSPERO prior to starting the review process (CRD42013004204) [12].

### Eligibility criteria for considering studies for this review

The eligibility criteria were:

- Types of studies: randomized controlled trial;
- Types of participants: children aged less than 18 years with asthma. We did not consider other kinds of allergic conditions;
- Types of interventions: vitamin D supplementation, without restrictions regarding dose (e.g., high or low), route of administration (e.g., oral, parenteral) or dosage interval (e.g., daily, weekly). The comparator was no vitamin D supplementation or placebo;
- Types of outcome measures: asthma related symptoms (e.g., nighttime awakenings, interference with normal activity, short-acting beta2-agonist use for symptom control), exacerbations requiring oral systemic corticosteroids or hospitalization, mortality, quality of life (measured using a validated instrument such as the Asthma Quality of Life Questionnaire (AQLQ)), and vitamin D related side effects (e.g., nausea/vomiting, constipation, loss of appetite).

We did not exclude studies based on language or date of publication.

### Search strategy

We designed the search strategy with the help of a medical librarian. The main search strategy consisted of searching the following electronic databases using the OVID interface from inception till February 2013: MEDLINE and EMBASE. The search combined terms for asthma, vitamin D, and pediatrics and included both free text words and medical subject heading. We did not use any search filter. The appendix provides the full details of the search strategies (see Additional file 1).

We used the following additional search strategies:

1. Search of the grey literature: theses and dissertations;
2. Search of the abstracts and proceedings from the following scientific meetings: American Thoracic Society (ATS), American College of Chest Physicians (ACCP), Pediatric Academic Societies, European Respiratory Society, European Society for Pediatric Research, American College of Allergy, Asthma & Immunology.
3. Review of references lists of included and relevant papers
4. Forward searching of included papers (ISI Web of Science)
5. Search of clinical trial registries
  - ClinicalTrials.gov http://clinicaltrials.gov/
  - International Standard Randomised Controlled Trial Number (ISRCTN) http://www.controlled-trials.com/isrctn/
  - Register EU Clinical Trials Register (EU-CTR) https://www.clinicaltrialsregister.eu
  - International Clinical Trial Registry Platform (ICTRP) http://apps.who.int/trialsearch/
6. Contact of authors of included studies inquiring about potentially eligible studies that we might have missed.

### Selection of studies

Two reviewers (LHA, MMF) screened in duplicate and independently the titles and abstracts of identified citations for potential eligibility. We obtained the full text for citations judged as potentially eligible by at least one of the 2 reviewers. The two reviewers then screened in duplicate and independently the full texts for eligibility. They used a standardized and pilot tested screening form and resolved disagreement by discussion. A senior team member (EAA) provided oversight.

### Data collection

The two reviewers (LHA, MMF) abstracted in duplicate and independently data from eligible studies. They used a standardized and pilot-tested screening form and detailed written instructions. They resolved disagreement by discussion. A senior team member (EAA) provided oversight. We calculated the agreement between the two authors for the assessment of trial eligibility using kappa statistic.

The data abstracted included the type of study and funding, the characteristics of the population, intervention, control, and outcomes assessed and statistical data.

### Assessment of risk of bias in included studies

The two reviewers assessed in duplicate and independently the risk of bias in each eligible study. They resolved disagreements by discussion or with the help of a third reviewer. According to recommendations outlined in the Cochrane Handbook [13], we used the following criteria for assessing the risk of bias in randomized studies:

- Inadequate sequence generation;
- Inadequate allocation concealment;
- Lack of blinding of participants, providers, data collectors, outcome adjudicators, and data analysts
- Incompleteness of outcome data;
- Selective outcome reporting, and other bias.

We graded each potential source of bias as high, low or unclear risk of bias.

### Data analysis and synthesis

All studies reported their outcomes as continuous data. For each trial and for each outcome, we calculated the mean difference when all trials used the same scale and the standardized mean difference when trials used different scales. We pooled the results of trials using a random-effects model. We tested results for homogeneity across trials using the I^2^ test and consider heterogeneity substantial if I^2^ was greater than 50%. For the meta-analysis of vitamin D levels, we converted values reported in nmol/l by Schou et al. to ng/ml [14].

The number of studies was too small to create inverted funnel plots in order to check for possible publication bias. Similarly, we did not conduct planned subgroup or sensitivity analyses due to the limited number of included studies. We interpreted SMDs using the following rules suggested by the Cochrane Handbook [13]:<0.40 represents a small effect size; 0.40 to 0.70 represents a moderate effect size; >0.70 represents a large effect size.

We assessed the quality of evidence by outcome using the GRADE methodology [15]. We produced a GRADE Summary of Findings table to summarize the statistical findings and quality of evidence by outcome.

## Results

### Search results

**Figure 1:**
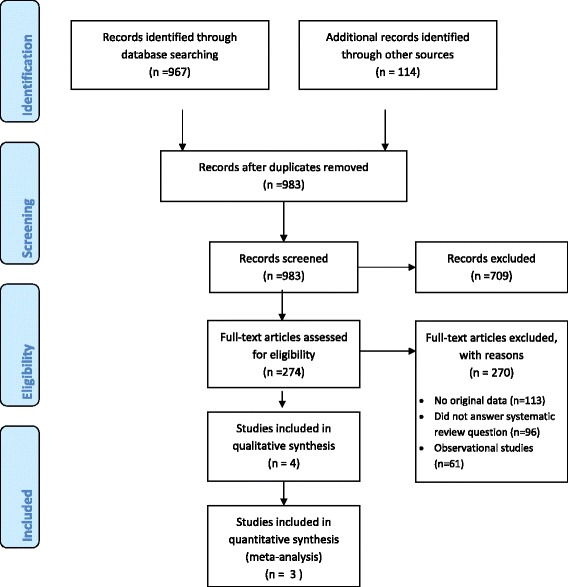
**PRISMA flow diagram.**

Figure 1 shows the study flow. The search strategy identified a total of 983 citations. Out of these, we assessed 274 full texts, of which we included 4 eligible studies [14,16-18]. The reasons for excluding the 270 full texts were as follows: 113 did not include original data, 96 did not answer our systematic review question, and 61 were observational studies. The agreement between the 2 reviewers for full text screening was high (kappa =0.94).We identified 12 ongoing trials assessing the effects of vitamin D in children with asthma symptoms (see Additional file 1 for more details).

### Included studies

Table 1 summarizes the characteristics of included studies. We could not include data from Lewis et al [18] in the quantitative analyses because not all necessary statistics (e.g., standard deviation) were reported. The four studies were published in English between 2003 and 2012. All four studies were randomized. Three of the studies had a parallel study design [16-18], while the remaining study (Schou [14]) had a crossover design. The numbers of participants included in the studies were 17 [14], 30 [18], 48 [17], and 54 [16], with a total of 149 participants included in this systematic review.

**Table 1:** **Characteristics of included studies**

**Study name**	**Study design/ funding**	**Participants**	**Intervention**	**Outcomes**	**Notes**
**Majak [** 16 **]** **2009**	● Randomized, double-blind, placebo-controlled	● 54 Patients sensitized only to house dust mites (HDM) as evidenced by a positive skin prick test.	● Intervention: Vitamin D3 Cholecalciferol, 1000 IU/week orally, single dose, for 1 year.	● Asthma symptoms measured at 3 and 12 months using a diary card (no validation of the diary card reported).	● Time frame: Sep 2005-April 2007
					● The study included a third placebo arm that we considered not relevant for this systematic review.
	Trial	● Age: 6–12 years (61% males)	● Control: No vitamin D3		
	● Funding: Medical University of Lodz, Poland	● Exclusion Criteria: FEV1 < 70%, any contraindication for SIT(Specific Immunotherapy), need for Budesonide (Inhaled Corticosteroid) dose of less than 400 mcg, or more than 800 mcg, any previous receiving of SIT, sensitization to allergens other than HDM, any discontinuation of SIT for any reason, maintenance dose of allergen extract not reached within 3 months of build-up phase of SIT, or missing more than one maintenance dose of allergen extract.	● Both arms received prednisone 20 mg For 3 month, and specific immunotherapy for 1 year.		
				● FEV1 at 3 and 12 month.	
				● Percentage reduction of median daily ICSs (Inhaled Corticosteroids) Dose at 3 and 12 months.	
				● Serum 25 hydroxyvitamin D3 levels (ng/ml) at 3 months.	
		● Mean serum 25 hydroxyvitamin D levels at baseline: 31.3 (SD: 3.4), 32.0 (SD: 3.1), for control and intervention groups, accordingly.			
		● Setting: Allergy clinic in Poland.			
**Lewis [** 18 **]** **2012**	● Pilot study. Randomized Controlled Trial	● 30 Patients diagnosed with chronic persistent asthma and on current daily controller asthma medication; all nonwhite.	● Intervention: vitamin D3 (Cholecalciferol) 1,000 IU, daily for 1 year.	● Asthma Control Test scores (ACT) (validated score [19]) at 6 month and 12 month.	● Time frame: 1 year.
			● Control: Placebo.		
	● Funding: LB595	● Age: 6-17 years			
				● FEV1 at 6 month and 12 month	
	State of Nebraska Tobacco Settlement	● Mean Asthma Control Test (ACT) Score at baseline was 17.8.			
				● Serum 25 hydroxyvitamin D3 levels (ng/ml) at 6 month and 12 month	
	Funds.	● Baseline vitamin D levels not reported			
		● Setting: Creighton University Medical Center.			
					Parameters were assessed at baseline in the winter, at 6 months later in the summer, and at 12 months later during the next winter.
**Schou [** 14 **]** **2003**	Randomized, double-blind, two-period crossover trial.	● 17 Patients: (14 boys)	● Intervention: Vitamin D3 (cholecalciferol) 15 μg (600 IU), vitamin A 1.5 mg, thiamine 3 mg, riboflavin 3 mg, nicotinamid 20 mg, ascorbic acid 75 mg, pyridoxine 2 mg, and panthotenic acid 8 mg, once daily in the morning, for four weeks.	● Asthma symptoms score (Developed by investigator; no validation of the score reported) at 4 weeks.	● Time frame: November-January (year not specified).
		● Age: 6-14 years.			
		● Mean height: 144.4 (104.8–176.2) cm			
	Funding: Not reported.	● Mean weight 38.8(16.8–72.6) kg			● Run-in and
		● Pre pubertal: 12 boys and 2 girls.			washout periods of 2 weeks and treatment periods of 4 weeks’ duration.
		● Treated with inhaled corticosteroid for at least one year before entering the study.		● FEV1 at 4 weeks.	
				● Use of Beta 2 agoinsts (Puffs/day) at 4 weeks.	
		● Baseline vitamin D levels not reported			
			● Control: Placebo.	● Serum 25 hydroxyvitamin D3 levels (ng/ml), mean level.	
		● Setting: Outpatient Children’s Clinic, in Randers, Denmark.	● Both arms received inhaled dry-powder budesonide 400 μg, daily, for four weeks.		
**Majak [** 17 **]** **2011**	● Randomized, double-blind, parallel-group trial.	● 48 Patients with newly diagnosed asthma and sensitive only to house dust mites	● Intervention: vitamin D3(cholecalciferol)500 IU.Dosage details not described.	● Asthma Therapy Assessment Questionnaire (ATAQ) every month up to six month (validated score [20]).	● Time frame: 6 months.
	● Funding: Medical University of Lodz, Poland.	● Mean age: 11.5 (5-18) years.			
		● Newly diagnosed asthma, sensitive only to House Dust Mite (HDM).	● Control: placebo.		
			● Both arms received budesonide 800 mg/d administered as a dry powder for six month.		
				● FEV1, mean of monthly measures	
				● Serum 25 hydroxyvitamin D3 levels (ng/ml), mean of a monthly measures.	
				● Number of Children with Asthma Exacerbations.	
		● Exclusion criteria included treatment with an oral, inhaled, or intranasal corticosteroid and supplementation with vitamin D during 6 months preceeding the trial, history of fractures in the last 2 years, immunotherapy, obesity (BMI > 30 Kg/m^2), and other chronic diseases.			
		● Mean serum 25 hydroxyvitamin D levels at baseline 35.1 (SD:16.9), 36.1			
		(SD:13.9), for control and intervention groups, accordingly.			
		● Setting: Allergy clinic in Poland.			

Two studies were conducted in an allergy clinic in Poland [16,17], while the other two were conducted in an outpatient children clinic in Denmark [14] and in USA [18]. Two studies included only house dust mite sensitized asthma patients [16,17], while the other two included patients with chronic asthma on daily asthma medications [14,18]. Only two studies reported mean baseline serum 25 hydroxyvitamin D levels [16,17]. These levels were mostly within normal limits [21]. One study excluded patients with severe asthma (FEV1 < 70%) [16].

The dose and duration of vitamin D supplementation varied across the included studies as follows: six weeks with 600 IU/day [14], six months with 500 IU/day [17], twelve months with 1,000 IU/day [18], and nineteen months with 1000 IU/week [16].

### Risk of bias in included studies

Table 2 summarizes the assessment of risk of bias in included studies. In terms of sequence generation, three reported adequate methods [14,16,17] while the fourth did not report on the method used. None of the studies reported on the method of allocation concealment. Three studies [14,16,17] reported using blinding, while the fourth study [18] did not. All studies reported number of participants with missing data; two had relatively high numbers of missing data: 11.7% in Schou et al. and 33.3% in Lewis et al. [14,18].

**Table 2:** **Assessment of risk of bias in included studies**

**Study name**	**Random sequence generation**	**Allocation concealment**	**Blinding**	**Intention to treat analysis**	**Completeness of data**	**Selective outcome reporting**	**Early stoppage of trial**
**Majak,** **[** 16 **]** **2009**	Low risk. “Patients were randomized according to a computer-generated stratified allocation schedule for intervention”	Unclear risk	Low risk.	Unclear risk.	Data for 2 patients (5%) missing	Low risk.	Low risk
			Probably patients, providers, data collectors and outcome assessors were blinded given the use of placebo	In the intent-to-treat analysis population excluded patients who received intervention for less than 2 months. Number of those excluded not reported		All outcomes listed in the trial registry and in the methods section are reported in the results section	Not stopped early for benefit
**Lewis,** **[** 18 **]** **2012**	Unclear risk	Unclear risk	Unclear risk	Unclear risk	High risk	Low risk	Low risk
	No details reported	No details reported	Probably no one blinded	No details reported	data for 15 patients (33.3%) missing	No published protocol but outcomes listed in the methods section are reported in the results section.	Not stopped early for benefit.
**Schou,** **[** 14 **]** **2003**	Low risk	Unclear risk	Low risk.	Unclear	Missing data: 2	Low risk	Low risk
	“Treatment order was allocated by means of a computerized randomization scheme”.	No details reported	Probably patients, providers, data collectors and outcome assessors were blinded given the use of placebo	No details reported	patients (11,7%).	No published protocol but outcomes listed in the methods section are reported in the results section.	Not stopped early for benefit.
**Majak, [** 17 **]** **2011**	Low risk.	Unclear	Low risk.	Unclear	Low risk.	Low risk	Low risk
	“Patients were randomized according to a computer-generated allocation schedule.”	No details reported	Probably patients, providers, data collectors and outcome assessors were blinded given the use of placebo	No details reported	No missing data.	No published protocol but outcomes listed in the methods section are reported in the results section.	Not stopped early for benefit

### Effects of interventions

#### Asthma symptoms

Three studies reported statistical data about the effect of vitamin D on asthma symptoms, using different scales [14,16,17]. While one study used a validated score [17,20], the other two used respectively a diary card [16] and a score without any evidence of validation reported [14]. As we were uncertain whether these different instruments are actually measuring the same outcome, we opted not to pool the results. While all three studies reported improvement in asthma symptoms in the vitamin D supplemented group study, there was no statistically significant difference between this group and the comparison/placebo groups [14,16,17]. The fourth study by Lewis et al. reported that Vitamin D supplementation did not affect the asthma symptom score [18]. The associated level of quality of evidence was judged to be very low due to risk of bias, heterogeneity and imprecision (see Table 3).

**Table 3:** **Summary of findings table**

**Vitamin D compared to No vitamin D for children with asthma**			
**Outcomes**	**No of participants (studies)** **follow up**	**Quality of the evidence** **(GRADE)**	**Anticipated effects**
**Asthma related symptoms**	116	⊕⊝⊝⊝	Heterogeneous and not definitive data, not pooled
Different instruments/scales	(3 studies)	**VERY LOW** ^1,2,3^	
	1.5-12 months	due to risk of bias, heterogeneity, imprecision	
**FEV1**	82	⊕ ⊕ ⊝⊝	The mean FEV1 in the intervention groups was
	(2 studies)	**LOW** ^3,4,5^	**0.54 lower** (5.28 lower to 4.19 higher)
	6-12 months	due to risk of bias, imprecision	
**Vitamin D levels**	116	⊕⊝⊝⊝	The mean vitamin D levels in the intervention groups was
	(3 studies)	**VERY LOW** ^1,6,7^	**6.6 higher** (0.6 lower to 13.8 higher)
	1.5-6 months	due to risk of bias, heterogeneity, imprecision	

GRADE Working Group grades of evidence.

**High quality:** Further research is very unlikely to change our confidence in the estimate of effect.

**Moderate quality:** Further research is likely to have an important impact on our confidence in the estimate of effect and may change the estimate.

**Low quality:** Further research is very likely to have an important impact on our confidence in the estimate of effect and is likely to change the estimate.

**Very low quality:** We are very uncertain about the estimate.

^1^None of the studies reported the methods of allocation concealment, and the use of intention to treat analysis. Schou 2003 had missing data for 12% of participants. ^2^High degree of unexplained heterogeneity with I2 = 86%.

^3^Wide confidence interval, including both values suggesting harms and values suggesting benefits.

^4^None of the studies reported the methods of allocation concealment, and the use of intention to treat analysis.

^5^Borderline degree of heterogeneity, I2 = 54%.

^6^High degree of unexplained heterogeneity with I2 = 97%.

^7^Wide confidence interval, including values suggesting no effect and values suggesting benefit.

### FEV-1

**Figure 2:**
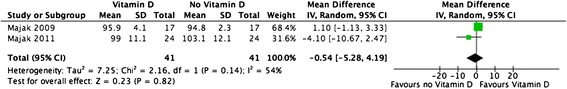
**Meta-analysis for FEV-1.**

Two of the included studies assessed post treatment FEV-1% predicted [16,17]. A meta-analysis resulted in a mean difference of 0.54% predicted, 95% CI (-5.28; 4.19) (See Figure 2). The level of heterogeneity was moderate (I^2^ 54%). We did not include a third study assessed in the meta-analysis because it expressed the outcome as a mean FEV-1 level and we could not obtain the data as FEV-1% predicted from the author. That study found no clinically or statistically significant difference between the two arms (2.08 (SD 0.12) versus 2.10 (SD 0.12); p = 0.60) [14]. Lewis et al. reported that Vitamin D supplementation did not affect FEV-1 [18]. The associated level of quality of evidence was judged to be low due to risk of bias and imprecision (see Table 3).

### Vitamin D levels

**Figure 3:**
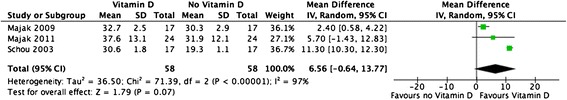
**Meta-analysis for vitamin D levels.**

Three of the included studies reported the effect on vitamin D levels [13,15,16]. A meta-analysis resulted in a mean difference of 6.56 ng/ml, 95% CI (-0.64; 13.77) (See Figure 3). The level of heterogeneity was high (I^2^ 97%). Lewis et al. reported that vitamin D levels in both groups increased significantly from baseline but did not differ significantly from each other at 6-month follow-up [17]. See Table 4 for reported details on Vitamin D dosage, supplementation duration used in each study, in addition to the interpretation of serum Vitamin D levels in each group of the included studies. The associated level of evidence was judged to be very low due to risk of bias, heterogeneity and imprecision (see Table 3).

**Table 4:** **Vitamin D dosage, duration, and serum level interpretation**

**Study name**	**Vitamin D (Dose-Duration)**	**Interpretation of serum 25(OH)D levels data as reported in each study**
**Majak,** **[** 16 **]** **2009**	1000 IU/week-nineteenth month	No significant changes were found between study groups
**Lewis,** **[** 18 **]** **2012**	1,000 IU/day-twelve month	No significant changes were found between study groups
**Schou,** **[** 14 **]** **2003**	600 IU/day-six weeks	Significantly higher levels were found during vitamin D supplementation period as compared to the levels of placebo period
**Majak,** **[** 17 **]** **2011**	500 IU/day-six month	25(OH)D serum levels were found insufficient in both study groups

### Other outcomes

Only one study reported on the outcome of acute asthma exacerbations [17]. Over a follow up period of over six month, the percentage of children who experienced asthma exacerbation was significantly lower in the Vitamin D group (17% versus 46%, p = 0.029). This quality of evidence could be judged as low, at best, given the high risk of bias and the imprecision associated with the very small number of events. None of the identified studies reported on the effects on mortality and quality of life, and adverse effects associated with vitamin D.

## Discussion

Our systematic review identified four randomized clinical trials assessing the effects of vitamin D supplementation in children with asthma. None of the identified studies reported on the effects on mortality, quality of life, or adverse effects associated with vitamin D supplementation. Meta-analysis neither confirmed nor ruled out beneficial effects of vitamin D supplementation on lung function and vitamin D levels. The associated quality of evidence was rated as very low or low due to risk of bias, heterogeneity and imprecision.

The limitations of this review are related to those of the identified evidence. Not only studies were at high risk of bias, but also too small to provide precise results. In addition, their results were heterogeneous for asthma symptoms and vitamin D level outcomes. Due to the limited number of studies, we could not conduct subgroup analyses (e.g., based on pre-treatment level of Vitamin D) to attempt to explain this heterogeneity. None of the studies reported on vitamin D adverse effects. However, the doses used are generally considered to be safe and unlikely to be associated with adverse effects [22].

This systematic review has a number of strengths. To our knowledge, this is the first systematic review assessing the effects of vitamin D supplementation in children with asthma. We used standard systematic review methodology in literature searching, study selection, data abstraction, risk of bias assessment, and quality of evidence rating. Also, we have identified 12 ongoing trials, making future updates of this systematic review likely to provide precise and accurate estimates of both benefits and harms of vitamin D supplementation in children with asthma. Those might also allow us to explore whether any effect modifiers such as pre-treatment level of Vitamin D can explain any heterogeneity of results.

## Conclusions

The major implication of our findings for clinical practice is that vitamin D cannot be considered for routine supplementation in children with asthma based on the currently available, at best, low quality evidence. Irrespectively, clinicians should consider vitamin D supplementation in children with low levels of vitamin D. However, our review does not address the question whether clinicians need to routinely test vitamin D levels in children with asthma.

Our findings have implications for future research. Future studies should be designed and executed in a way to minimize the risk of bias, and be reported clearly and comprehensively. Trials also need to be adequately powered to assess with precision the effects on the most important patient outcomes, including exacerbation, hospital admission, symptoms, quality of life, and adverse effects.

## Additional file

Additional file 1:
**Appendix 1- detailed search strategies.** Appendix 2 - Ongoing clinical trials assessing the effects of vitamin D supplementation in children with asthma.
