# Supplementary material for: Vitamin D supplementation in children with asthma: a systematic review and meta-analysis
Source: BMC Res Notes. 2015 Feb 3;8:23. doi: 10.1186/s13104-014-0961-3 (PMC4328422; doi:10.1186/s13104-014-0961-3)
Supplement: Additional file 1: — Appendix 1- detailed search strategies. Appendix 2 - Ongoing clinical trials assessing the effects of vitamin D supplementation in children with asthma. [file 13104_2014_961_MOESM1_ESM.doc]

## Additional file 1

Appendix 1**- detailed search strategies**

- MEDLINE: [(exp vitamin d/ or expcholecalciferol/ or exphydroxycholecalciferols/ or expcalcifediol/ or expdihydroxycholecalciferols/ or expcalcitriol/ or exp 24,25-dihydroxyvitamin d 3/ or expergocalciferols/ or expdihydrotachysterol/ or exp 25-hydroxyvitamin d 2/)OR (vitamin D or vitamin D2 or Vitamin D3 or ergocalciferol* or alfacacidol* or alfa-cacidol* or calcitriol* or cholecalciferol* or calciol* or calciferol* or hydroxycholecalciferol* or hydroxy-cholecalciferol* or dihydroxycholecalciferol* or dihydroxy-cholecalciferol* or 24,25-dihydroxyvitamin d3 or 24,25-dihydroxy-vitamin d3 or dihydrotachysterol* or dihydro-tachysterol* or 25-hydroxyvitamin d2 or 25-hydroxy-vitamin d2 or vitamin D deficien*)OR(exp vitamin d deficiency/ or exposteomalacia/ or exp rickets/)] AND[(exp asthma/ or exp bronchial hyperreactivity/ or exp respiratory hypersensitivity/ or expalveolitis, extrinsic allergic/ or expaspergillosis, allergic bronchopulmonary/)OR((bronch* or respirat*) adj3 (hyperreactiv* or hyper-reactiv* or hypersensitiv* or hyper-sensitiv*))OR((asthma* or wheezing*).mp.OR((reactiv* adj3 airway*).mp.OR(exp anti-asthmatic agents/ or exp bronchodilator agents/)OR(anti asthmatic* or anti-asthmatic* or bronchodilator* or broncho-dilator*).mp.] AND[(exp pediatrics/ or exp neonatology/ or exp perinatology/)OR(exp adolescent/ or exp child/ or exp child, preschool/ or exp infant/ or exp infant, newborn/ or exp infant, low birth weight/ or exp infant, small for gestational age/ or exp infant, very low birth weight/ or exp infant, postmature/ or exp infant, premature/ or exp infant, extremely premature/)OR(exp Infant, Newborn, Diseases/)OR(infant* or child* or adolescen* or pediatric* or paediatric* or newborn* or new-born* or baby or babies or neonat* neo-nat* or toddler* or preschool* or pre-school* or teenage* or teen-age*).mp.]
- EMBASE: [(exp vitamin d/ or expcholecalciferol/ or exphydroxycholecalciferols/ or expcalcifediol/ or expdihydroxycholecalciferols/ or expcalcitriol/ or exp 24,25-dihydroxyvitamin d 3/ or expergocalciferols/ or expdihydrotachysterol/ or exp 25-hydroxyvitamin d 2/ or exp vitamin d deficiency/ or exposteomalacia/ or exp rickets/)OR (vitamin D or vitamin D2 or Vitamin D3 or ergocalciferol* or alfacacidol* or alfa-cacidol* or calcitriol* or cholecalciferol* or calciol* or calciferol* or hydroxycholecalciferol* or hydroxy-cholecalciferol* or dihydroxycholecalciferol* or dihydroxy-cholecalciferol* or 24,25-dihydroxyvitamin d3 or 24,25-dihydroxy-vitamin d3 or dihydrotachysterol* or dihydro-tachysterol* or 25-hydroxyvitamin d2 or 25-hydroxy-vitamin d2 or vitamin D deficien*)]AND[(exp asthma/ or exp bronchial hyperreactivity/ or exp respiratory hypersensitivity/ or expalveolitis, extrinsic allergic/ or exp allergic bronchopulmonary/ or exp anti-asthmatic agents/ or exp bronchodilator agents/)OR((bronch* or respirat*) adj3 (hyperreactiv* or hyper-reactiv* or hypersensitiv* or hyper-sensitiv*))OR(asthma* or wheezing*)OR(reactiv* adj3 airway*)OR(anti asthmatic* or anti-asthmatic* or bronchodilator* or broncho-dilator*)]AND[(exp pediatrics/ or exp neonatology/ or exp perinatology/ or exp adolescent/ or exp child/ or exp child, preschool/ or exp infant/ or exp infant, newborn/ or exp infant, low birth weight/ or exp infant, small for gestational age/ or exp infant, very low birth weight/ or exp infant, postmature/ or exp infant, premature/ or exp infant, extremely premature/ or exp Infant, Newborn, Diseases/)OR(infant* or child* or adolescen* or pediatric* or paediatric* or newborn* or new-born* or baby or babies or neonat* neo-nat* or toddler* or preschool* or pre-school* or teenage* or teen-age*)]

Appendix 2 - **Ongoing clinical trials assessing the effects of vitamin D supplementation in children with asthma**

| **Title; ID no.*** | **Type/Design** | **Study Population** | **Treatment Arms** |
| --- | --- | --- | --- |
| Vitamin D effects in asthma; IRCT201302079608N1 | Randomized Controlled Trial (RCT) | 10-50 years old asthmatics. | Intervention arm: Inhaled corticosteroid, and Vitamin D.  Control arm: Inhaled corticosteroid. |
| Randomized, multi-centre, double-blind, placebo-controlled trial of vitamin d supplementation in adult and adolescent; ISRCTN07270894 | Multicentre randomized interventional prevention trial | 12-80 years old asthmatics. | Intervention arm: Dietary supplement of cholecalciferol and miglyol oil  Control arm: Dietary supplement of miglyol oil |
| Effects of the vitamin D administration on respiratory functions in pediatric patients suffering from effort asthma; 2011-000762-35 | RCT | 6-14 years old asthmatics. | Intervention arm: Oral solution of vitamin D.  Control arm: Placebo |
| Vitamin D and atopic allergy; 2006-000359-16 | RCT | 0-6 years old healthy children. | Intervention arm: Oral solution of vitamin D and vitamin A.  Control arm: Placebo |
| Correction of Vitamin D in Asthma Control; NCT01395589 | RCT | 1-14 years with moderate to severe asthma and proved to be vitamin D deficient by serum level | Two comparators arms, arm 1: Oral solution of vitamin D.  Arm 2: Intramuscular form of vitamin D. |
| Vitamin D for the Treatment of Severe Asthma; NCT00712205 | RCT | >18 years old asthmatics. | Intervention arm: calcitriol  Control arm: placebo |
| Study of the Effect of Vitamin D as an Add-on Therapy to Corticosteroids in Asthma (VIDA); NCT01248065 | RCT | >18 years old asthmatics. | Intervention arm: Low dose inhaled corticosteroid and Vitamin D.  Placebo arm: Low dose inhaled corticosteroid and placebo |
| Vitamin D, Steroids, and Asthma in African American Youth (AsthMaP2); NCT01647399 | Prospective observational study | 6 -20 years old asthmatics. | - |
| Vitamin and Asthma Study; NCT01447173 | RCT | 7-17 years old asthmatics. | Intervention arm: 2000 IU vitamin D.  Control arm:  400 IU Vitamin D. |
| The Effect of Vitamin D Replacement on Airway Reactivity, Allergy and Inflammatory Mediators in Exhaled Breath Condensate in Vitamin D Deficient Asthmatic Children; NCT01287455 | RCT | 6-18 years old asthmatics. | Intervention arm: vitamin D.  Control arm: placebo. |
| DO IT Trial: Vitamin D Outcomes and Interventions In Toddlers; NCT01419262 | RCT | 1-5 years old healthy children. | Intervention arm: 2000 IU vitamin D  Control arm: 400 IU vitamin D |
| Vitamin D for Sickle-cell Respiratory Complications; NCT01443728 | RCT | 3 -20 years diagnosed with sickle cell disease. | Intervention arm: Oral vitamin D3, 100,000 IU.  Control arm: Oral vitamin D3 12,000 IU. |
